# Multitrait Evaluation of Drought Tolerance at the Seedling Stage in Advanced Backcross Germplasm Derived from Sugarcane × *Tripidium arundinaceum*

**DOI:** 10.3390/plants15182813

**Published:** 2026-09-14

**Authors:** Jiarui Liu, Yu Zhang, Yan Ye, Bo Yu, Chunxia Cheng, Yiting Liao, Chaohua Huang, Jiayun Wu, Xinwang Zhao, Zuhu Deng

**Affiliations:** 1 National Engineering Research Center for Sugarcane, Key Laboratory of Sugarcane Biology and Genetic Breeding, Ministry of Agriculture and Rural Affairs, Fujian Agriculture and Forestry University, Fuzhou 350002, China; jerryliu0273@163.com (J.L.);; 2 Institute of Nanfan & Seed Industry, Guangdong Academy of Science, Guangzhou 510316, China

**Keywords:** sugarcane, *Tripidium arundinaceum*, drought stress, drought tolerance, comprehensive evaluation

## Abstract

Drought is a major abiotic constraint on sugarcane production, but systematic multitrait evaluation of drought tolerance remains limited in genetically complex advanced backcross progenies derived from sugarcane × *Tripidium arundinaceum*. In this study, 100 genotypes, including 93 BC_4_F_1_ or BC_5_F_1_ progenies and seven parental or control genotypes, were evaluated under a 20 d progressive drought treatment in pots at the seven-leaf stage. Sixteen traits related to root architecture, leaf water status, gas exchange, PSII photochemistry, antioxidant activity, and lipid peroxidation were measured. Drought significantly reduced TRL, RSA, RV, RWC, Pn, Gs, Tr, ΦPSII, and ETR and significantly increased SOD activity and MDA content. Twelve traits with relatively clear and biologically interpretable relationships with drought tolerance were integrated using membership functions and trait weights to calculate an integrated D value. D values ranged from 0.078 to 0.516, and 19, 45, and 36 genotypes were classified into groups with relatively high, moderate, and low drought tolerance within this germplasm collection, respectively. Genotypes 55-109, YCE07-65, 55-43, 211-172, and 91-74 had the highest integrated D values. Within the integrated evaluation framework, higher D values were most strongly associated with RSA, RV, TRL, Pn, ΦPSII, and ETR, while PC1 was strongly correlated with the D value (*r* = 0.893). Maintenance of root growth and photosynthetic function therefore represented major phenotypic features associated with overall drought tolerance under the present experimental conditions. These features may provide useful criteria for screening drought-tolerant germplasm, but their utility requires validation in independent drought experiments and field environments.

## 1. Introduction

Sugarcane (*Saccharum* spp.) is an important crop for sugar and bioenergy production [1,2,3]. Water deficit is a major constraint on crop productivity, affecting plant water acquisition, stomatal conductance, and photosynthetic carbon assimilation, with the magnitude of these effects varying among genotypes and environmental conditions [4,5,6,7]. Root system architecture and hydraulic regulation play important roles in plant acclimation to declining soil water availability, while coordinated adjustments in stomatal behavior and photosynthetic function help regulate the balance between water conservation and carbon gain under drought [8,9]. In sugarcane, field studies have revealed substantial genotypic variation in stalk yield, dry matter accumulation, stomatal conductance, leaf functional traits, and photosynthetic performance under water-limited conditions [10,11,12]. Therefore, comprehensive evaluation of drought tolerance across diverse sugarcane materials and the identification of superior drought-tolerant germplasm are important for sugarcane breeding.

Drought tolerance is a complex trait involving multiple physiological and biochemical processes, including water acquisition and transport, stomatal regulation, maintenance of photosynthetic function, osmotic adjustment, and antioxidant defense [5,13]. The contribution of individual traits to drought adaptation can vary with genotype, stress intensity, developmental stage, and environmental conditions, making it difficult to characterize overall drought tolerance using any single indicator [14,15]. Consequently, multitrait evaluation approaches that integrate morphological, physiological, and yield-related responses have been increasingly used to assess drought tolerance and identify contrasting germplasm in crop species [16]. Such approaches have also been applied in sugarcane. Xu et al. evaluated drought tolerance in sugarcane germplasm using multiple traits [17], whereas Deng et al. classified drought tolerance in BC_3_F_1_ progeny derived from sugarcane × *Erianthus arundinaceus* [18]. However, studies integrating root system architecture, leaf water status, gas exchange, PSII photochemical performance, and antioxidant responses remain limited in advanced backcross populations derived from *Tripidium arundinaceum*, which have higher backcross generations and more complex genetic backgrounds.

Crop wild relatives provide valuable genetic diversity for broadening the genetic base of cultivated crops and introducing useful alleles for adaptation to environmental stresses [19,20]. This is particularly relevant to modern sugarcane, for which the utilization of related wild species provides an important source of novel genetic variation for crop improvement [21]. *Tripidium arundinaceum* (formerly *Erianthus arundinaceus*) is an important wild relative of sugarcane with high biomass production, strong ratooning and root-forming ability, and broad adaptation to adverse environments [22,23]. Cytogenetic studies have demonstrated that *T. arundinaceum*-derived chromosomes can be introgressed into the sugarcane genetic background through intergeneric hybridization and successive backcrossing, with chromosome transmission and intergeneric translocations documented across several backcross generations [24,25,26,27]. Recent physiological studies showed that *T. arundinaceum* and its intergeneric hybrids have relatively high intrinsic water-use efficiency, while large-scale germplasm evaluation revealed substantial genetic variation in drought-related physiological traits [28,29]. In addition, recent genomic analyses identified drought-responsive genes and genomic variation associated with climatic adaptation in *T. arundinaceum* [30]. However, systematic multitrait evaluation of drought tolerance remains limited in advanced sugarcane × *T. arundinaceum* backcross populations.

In this study, BC_4_F_1_ and BC_5_F_1_ advanced backcross populations derived from sugarcane × *T. arundinaceum* crosses were subjected to 20 d of progressive drought treatment under pot conditions. At the end of the treatment, traits related to root system architecture, leaf water status, gas exchange, PSII photochemical activity, antioxidant responses, and membrane damage were measured. Drought tolerance was evaluated using drought-tolerance coefficients, membership function analysis, and an integrated D value, while correlation analysis and principal component analysis (PCA) were used to identify traits closely associated with overall drought tolerance. The study aimed to identify drought-tolerant germplasm, characterize the major phenotypic differences in drought tolerance among advanced backcross progenies, and assess the potential value of *T. arundinaceum*-derived germplasm in sugarcane breeding for drought tolerance.

## 2. Results

### 2.1. Drought Stress Alters Root System Architecture and Leaf Water Status

**Figure 1:**
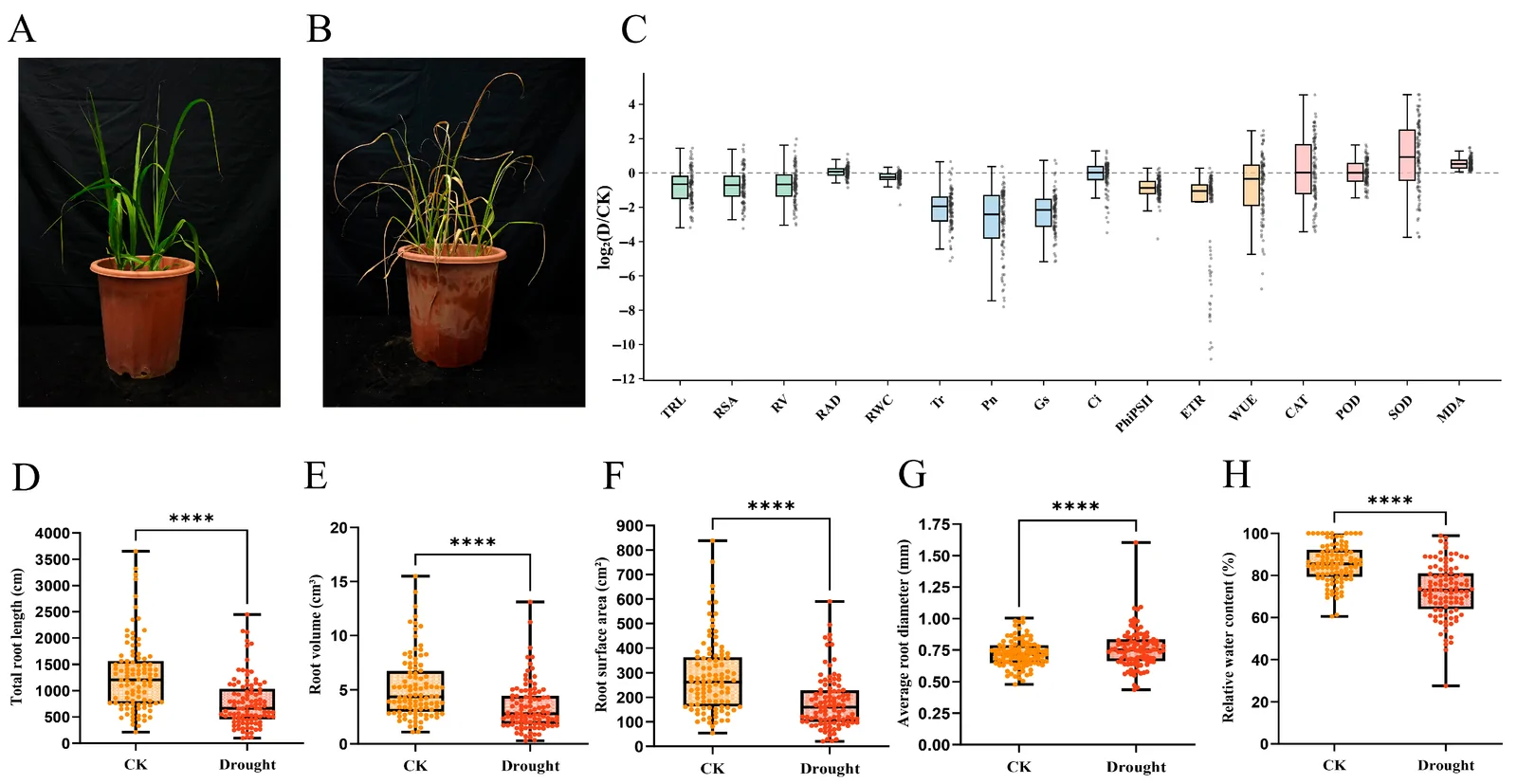
Effects of drought stress on plant phenotype, root morphology, leaf water status, and drought-tolerance coefficients of multiple traits in sugarcane. (**A**) Phenotype of representative plants under well-watered control (CK) conditions; (**B**) phenotype of representative plants after 20 d of drought treatment; (**C**) distributions of log_2_ (D/CK) values for 16 drought-response traits across 100 genotypes, where D and CK represent values under drought and well-watered control conditions, respectively, and the dashed horizontal line indicates log_2_ (D/CK) = 0; (**D**–**H**) distributions of total root length (TRL), root volume (RV), root surface area (RSA), root average diameter (RAD), and relative water content (RWC) under CK and drought conditions. The 16 traits included TRL, RSA, RV, RAD, RWC, transpiration rate (Tr), net photosynthetic rate (Pn), stomatal conductance (Gs), intercellular CO_2_ concentration (Ci), effective quantum yield of PSII photochemistry (ΦPSII), electron transport rate (ETR), water-use efficiency (WUE), catalase (CAT) activity, peroxidase (POD) activity, superoxide dismutase (SOD) activity, and malondialdehyde (MDA) content. In the boxplots, boxes indicate the interquartile range (25th–75th percentiles), center lines indicate medians, and each point represents the mean of three biological replicates for one genotype. Statistical significance represents the treatment main effect from a two-factor linear model including genotype, treatment, and genotype × treatment interaction. **** *p* < 0.0001.

After 20 d of drought treatment, plants showed pronounced leaf rolling, wilting, and yellowing, whereas control plants generally maintained normal growth (Figure 1A,B), indicating that the imposed drought treatment effectively induced a water-deficit response. During the treatment period, soil volumetric water content (VWC) in the well-watered controls was maintained at approximately 70–80%, whereas VWC in the drought-treated pots progressively declined to approximately 25% by day 20. No plant mortality was observed in either the well-watered control or drought-treated plants after 20 d of treatment. After log_2_ transformation of the drought-to-control ratios (D/CK) for the 16 traits, total root length (TRL), root surface area (RSA), root volume (RV), and relative water content (RWC) were predominantly distributed below zero, whereas root average diameter (RAD) was centered around zero with a slight shift toward positive values (Figure 1C).

At the population level, drought treatment significantly affected TRL, RSA, RV, RAD, and RWC (Figure 1D–H; all treatment main effects, *p* < 0.0001; Table S5). TRL, RSA, and RV decreased under drought, with mean D/CK ratios of 0.7195, 0.7395, and 0.7905, respectively (Table 1). RAD showed a relatively small increase, with a mean D/CK ratio of 1.0695, whereas RWC decreased, with a mean D/CK ratio of 0.8550. Overall, drought markedly reduced root system size and leaf water status at the population level, while RAD showed a small contrasting increase.

### 2.2. Drought Stress Suppresses Leaf Gas Exchange and PSII Photochemical Performance

**Figure 2:**
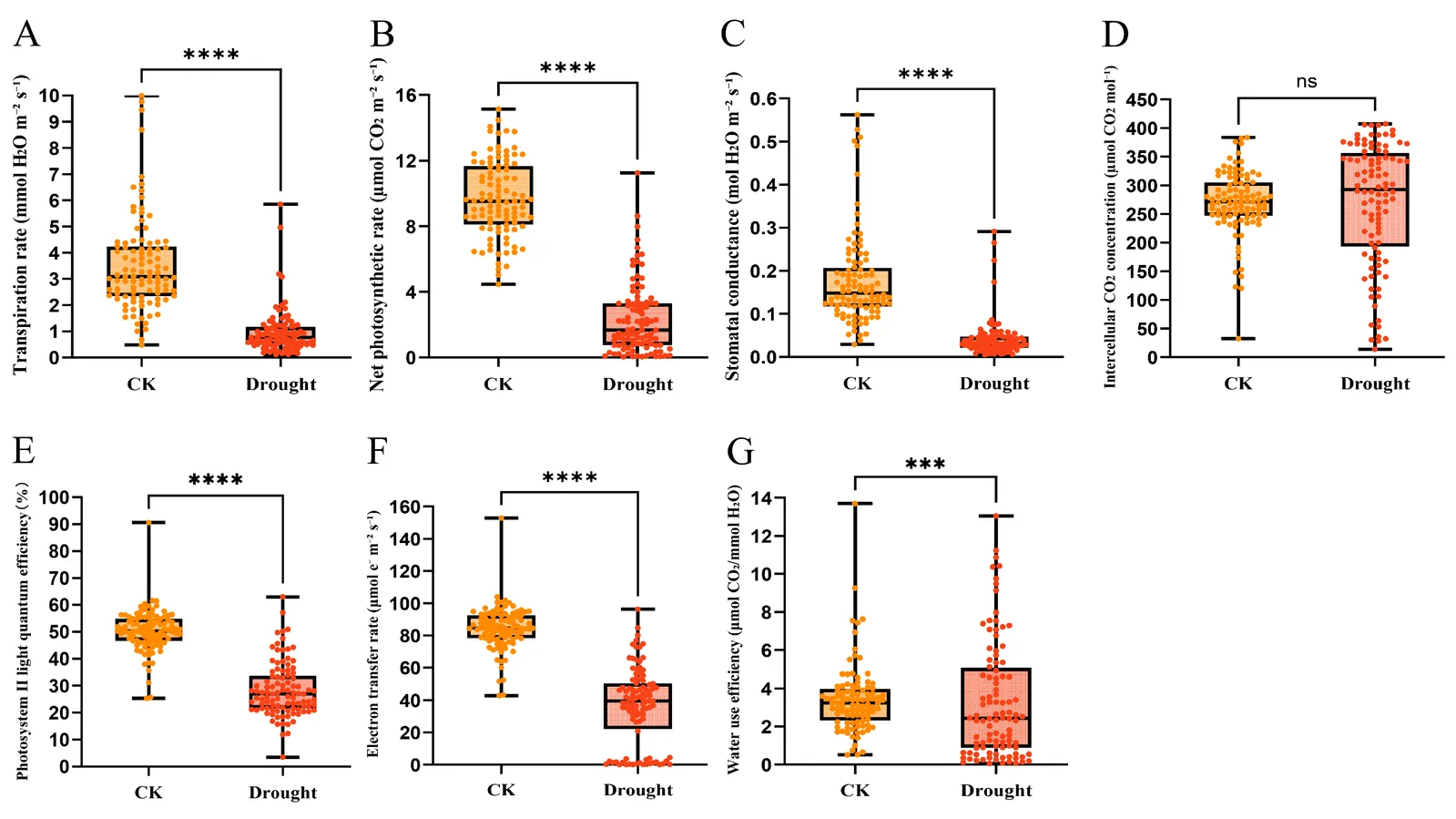
Effects of drought stress on leaf gas exchange and photosynthetic function in sugarcane. (**A**) Transpiration rate (Tr); (**B**) net photosynthetic rate (Pn); (**C**) stomatal conductance (Gs); (**D**) intercellular CO_2_ concentration (Ci); (**E**) effective quantum yield of PSII photochemistry (ΦPSII); (**F**) electron transport rate (ETR); and (**G**) water-use efficiency (WUE). Boxes indicate the 25th–75th percentiles, center lines indicate medians, and each point represents the mean of three biological replicates for one genotype. CK and Drought denote the well-watered control and drought treatment, respectively. Statistical significance indicates the treatment main effect from the two-factor linear model including genotype, treatment, and genotype × treatment interaction. *** *p* < 0.001; **** *p* < 0.0001; ns, not significant (*p* > 0.05).

Gas-exchange parameters showed some of the largest relative decreases among the measured traits. Drought treatment significantly affected transpiration rate (Tr), net photosynthetic rate (Pn), and stomatal conductance (Gs) (Figure 2A–C; all treatment main effects, *p* < 0.0001; Table S5). Tr, Pn, and Gs decreased under drought, with mean D/CK ratios of 0.2977, 0.2427, and 0.2722, respectively (Table 1). Pn showed the greatest relative decrease among the three gas-exchange traits, while Tr and Gs were also markedly reduced at the population level.

Intercellular CO_2_ concentration (Ci) showed no significant overall treatment effect (Figure 2D; *p* = 0.4900; Table S5), with a mean D/CK ratio of 1.0059 (Table 1). Ci nevertheless showed a broad distribution under drought, reflecting substantial variation in response among genotypes. Drought treatment also significantly affected the effective quantum yield of PSII photochemistry (ΦPSII) and electron transport rate (ETR) (Figure 2E,F; both treatment main effects, *p* < 0.0001; Table S5). Both traits decreased under drought, with mean D/CK ratios of 0.5769 and 0.4440, respectively (Table 1), indicating marked suppression of PSII photochemical performance. Instantaneous water-use efficiency (WUE) showed a statistically significant but small overall treatment effect (Figure 2G; *p* = 0.0003, partial η^2^ = 0.032; Table S5) while exhibiting substantial variation among genotypes. Overall, drought markedly reduced gas exchange and PSII photochemical performance at the population level, whereas Ci showed no significant average treatment effect and the overall treatment effect on WUE was relatively small.

### 2.3. Drought Stress Alters Antioxidant Enzyme Activities and Increases Lipid Peroxidation

**Figure 3:**
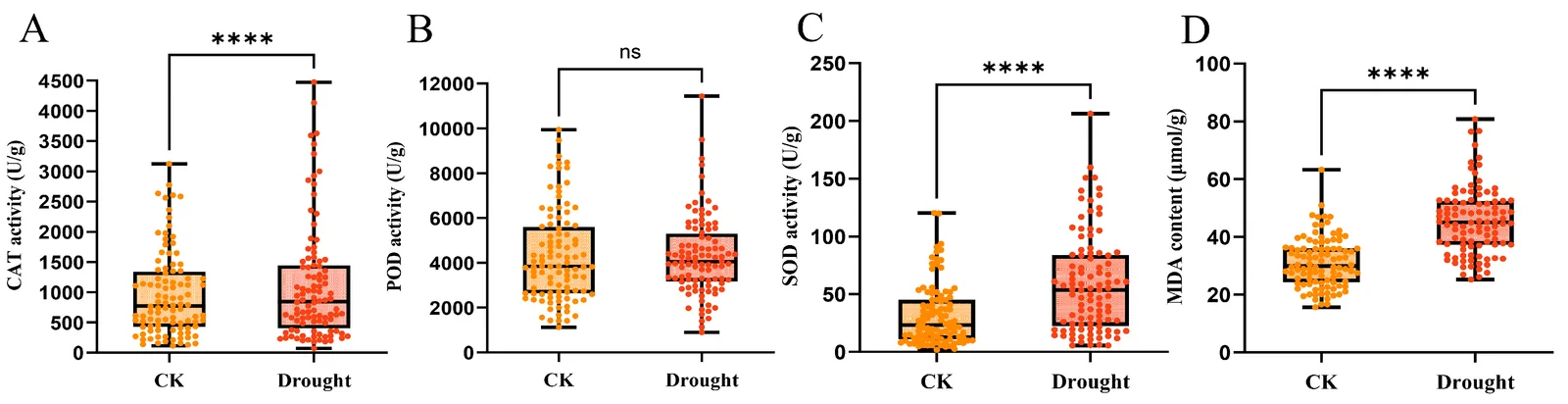
Effects of drought stress on antioxidant enzyme activities and lipid peroxidation in sugarcane leaves. (**A**) Distribution of catalase (CAT) activity under well-watered control (CK) and drought conditions; (**B**) distribution of peroxidase (POD) activity; (**C**) distribution of superoxide dismutase (SOD) activity; and (**D**) distribution of malondialdehyde (MDA) content. Boxes indicate the 25th–75th percentiles, center lines indicate medians, and each point represents the mean of three biological replicates for one genotype. CK denotes the well-watered control, and Drought denotes the drought treatment. Statistical significance indicates the treatment main effect from the two-factor linear model including genotype, treatment, and genotype × treatment interaction. **** p < 0.0001; ns, not significant (*p* > 0.05).

The effects of drought stress on antioxidant enzyme activities varied among enzymes. Catalase (CAT) activity showed a significant overall treatment effect (Figure 3A; *p* < 0.0001; Table S5), whereas peroxidase (POD) activity showed no significant overall treatment effect (Figure 3B; *p* = 0.9318; Table S5). The mean D/CK ratios of CAT and POD were 2.2983 and 1.1448, respectively (Table 1). CAT responses showed a broad distribution among genotypes, indicating substantial variation in the magnitude of the response. Superoxide dismutase (SOD) activity was also significantly affected by drought (Figure 3C; *p* < 0.0001; Table S5) and increased at the population level, with a mean D/CK ratio of 4.0437 (Table 1). MDA content showed a significant treatment effect (Figure 3D; *p* < 0.0001; Table S5) and increased under drought, with a mean D/CK ratio of 1.5046 (Table 1). Overall, drought significantly affected CAT and SOD activities and increased MDA accumulation, whereas POD showed no significant average treatment effect.

### 2.4. Multitrait Analysis Reveals Substantial Genotypic Variation in Drought Responses

Two-factor analysis of the original pot-level data detected significant genotype × treatment interactions for all 16 measured traits (all *p* < 0.0001; Table S5), indicating that the magnitude and/or direction of drought-induced changes differed significantly among genotypes. These interaction effects provided statistical support for the substantial genotypic heterogeneity observed in the drought-response profiles.

**Figure 4:**
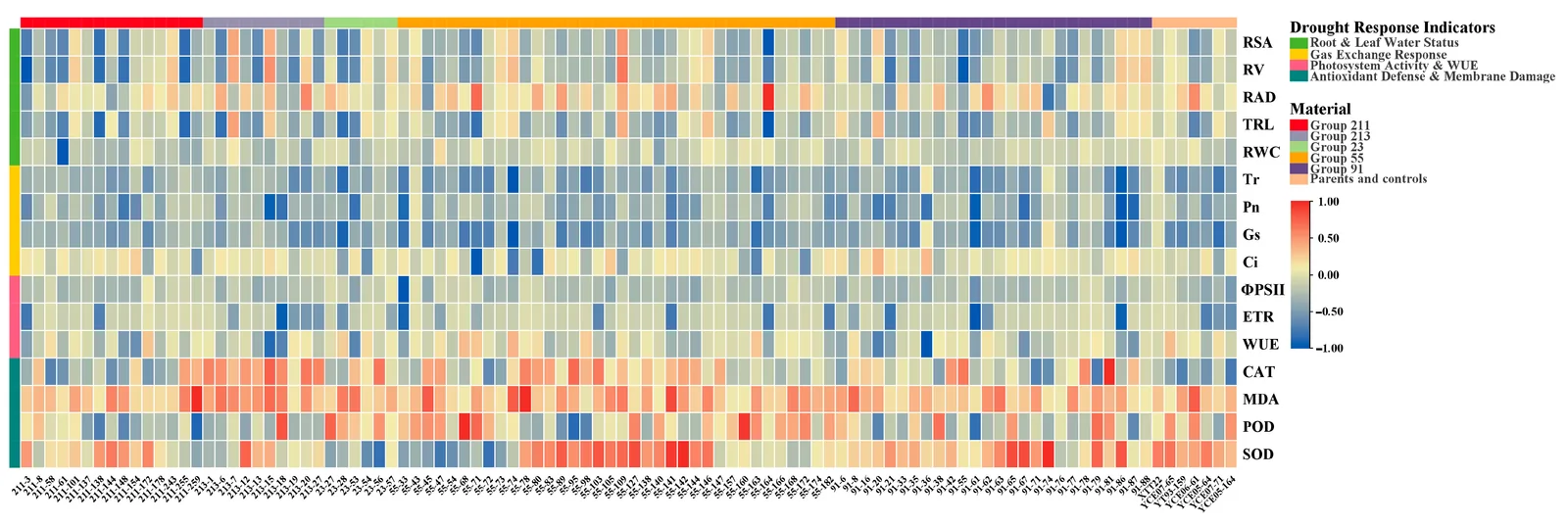
Normalized heatmap of 16 drought-response traits across 100 sugarcane genotypes. The heatmap was generated from log_2_ (D/CK) values calculated from the mean of three biological replicates for each genotype under each treatment and subsequently normalized by the maximum absolute value of each trait. Columns and rows represent genotypes and traits, respectively, with warm and cool colors indicating higher and lower normalized values. The top annotation bar indicates the origin of each genotype, and the left annotation bar indicates the functional category of each trait. RSA, root surface area; RV, root volume; RAD, root average diameter; TRL, total root length; RWC, relative water content; Tr, transpiration rate; Pn, net photosynthetic rate; Gs, stomatal conductance; Ci, intercellular CO_2_ concentration; ΦPSII, effective quantum yield of PSII photochemistry; ETR, electron transport rate; WUE, water-use efficiency; CAT, catalase activity; MDA, malondialdehyde content; POD, peroxidase activity; SOD, superoxide dismutase activity.

The heatmap based on maximum-absolute-value-normalized log_2_ (D/CK) values for the 16 traits showed distinct response patterns among genotypes in root system architecture, leaf water status, gas exchange, PSII photochemical performance, water-use efficiency, antioxidant enzyme activities, and MDA accumulation (Figure 4). The root-related traits TRL, RSA, RV, and RAD, together with RWC, showed both positive and negative responses among genotypes. Tr, Pn, Gs, ΦPSII, and ETR showed negative responses in most genotypes, with decreases in Pn, Gs, and ETR being particularly widespread, indicating that gas exchange and photosynthetic electron transport were among the physiological processes most strongly affected by drought. In contrast, Ci and WUE showed bidirectional responses, with both increases and decreases observed among genotypes.

Antioxidant- and oxidative damage-related traits also showed substantial genotypic variation. SOD activity and MDA content increased in most genotypes, indicating widespread induction of SOD activity and increased lipid peroxidation under drought. In contrast, CAT and POD responses varied in both direction and magnitude among genotypes. Distinct response patterns were observed both among populations of different origins and within individual populations, consistent with the significant genotype × treatment interactions and indicating that drought responses in the tested germplasm were strongly genotype-dependent.

### 2.5. Integrated D Value Evaluation Reveals Substantial Variation in Drought Tolerance Among Genotypes

Among the 16 drought-response traits measured in this study, 12 traits showing relatively consistent directional responses and representing root growth, leaf water maintenance, photosynthetic function, antioxidant defense, and oxidative damage were selected for calculation of the integrated D value. Ci, Gs, Tr, and RAD were excluded because their relationships with drought tolerance are not consistently monotonic. These four traits were retained for interpretation of drought responses but were not included in the integrated drought-tolerance score.

**Figure 5:**
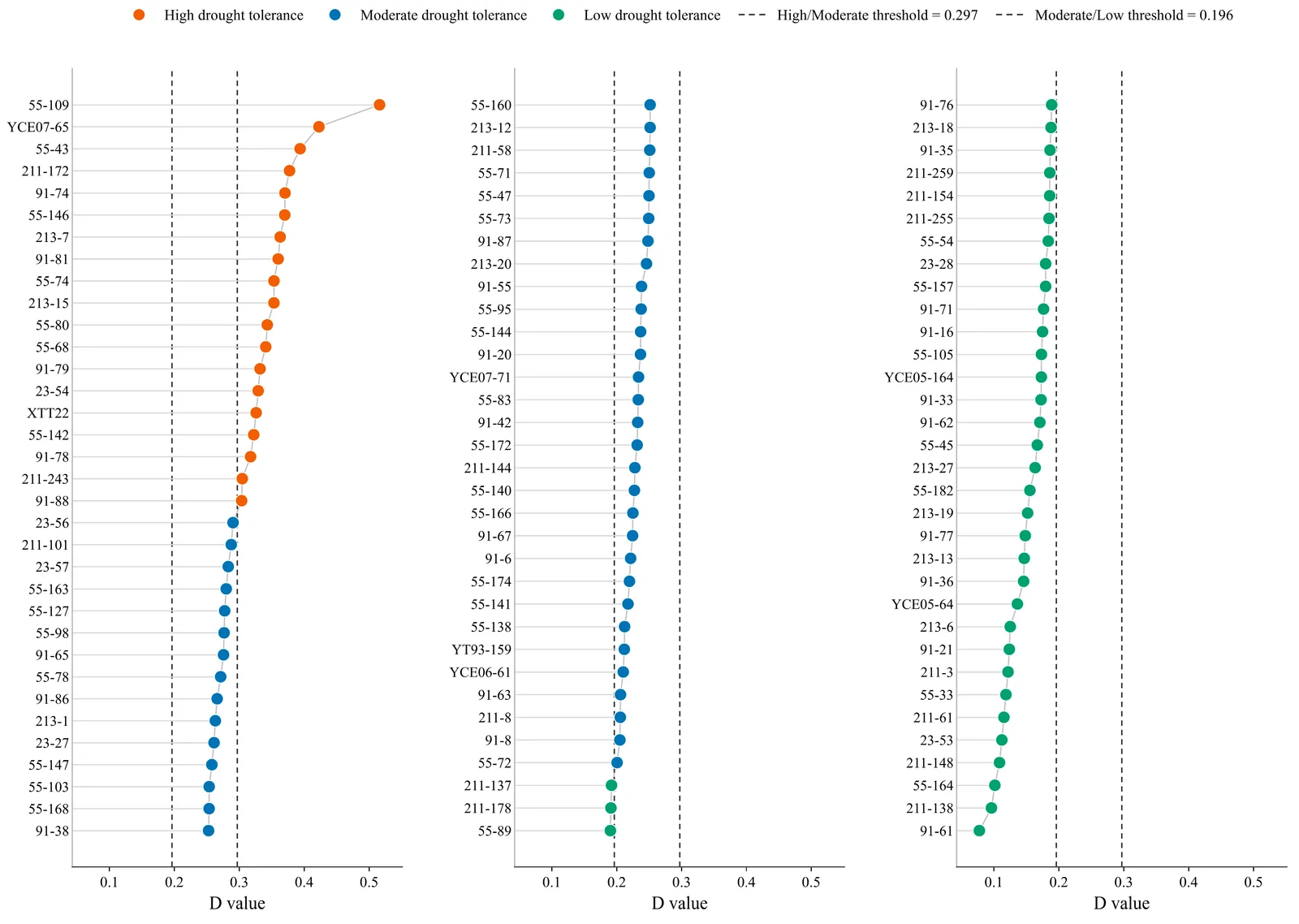
Ranking and classification of sugarcane genotypes for drought tolerance based on the integrated D value. Integrated D values were calculated for each genotype using 12 selected evaluation traits, and the genotypes were ranked in descending order of D value. For clarity, all genotypes are displayed across three panels according to their ranking. The *x*-axis shows the integrated D value, and the *y*-axis shows genotype names. Based on the cluster-derived thresholds of 0.297 and 0.196, the genotypes were divided into groups with relatively high, moderate, and low drought tolerance within the present germplasm collection. Orange, blue, and green circles indicate genotypes in the high-, moderate-, and low-drought-tolerance groups, respectively. The two vertical dashed lines indicate the thresholds between the high- and moderate-drought-tolerance groups (D = 0.297) and between the moderate- and low-drought-tolerance groups (D = 0.196), respectively.

Based on the 12 selected traits, integrated D values for the 100 genotypes ranged from 0.078 to 0.516 (Figure 5). Using the cluster-derived thresholds of 0.297 and 0.196, the genotypes were classified into groups with relatively high, moderate, and low drought tolerance within the present germplasm collection, containing 19, 45, and 36 genotypes, respectively. Genotype 55-109 had the highest D value (0.516), followed by YCE07-65 (0.423) and 55-43 (0.394), while 211-172 and 91-74 also ranked among the top five. The drought-tolerant control XTT22 had a D value above 0.297 and was assigned to the high-drought-tolerance group, consistent with its known drought-tolerant performance and providing an internal reference for the grouping (Table 2). Genotypes 55-146, 213-7, 91-81, 55-74, and 213-15 were also classified into the high-drought-tolerance group. At the other end of the ranking, 91-61 had the lowest D value (0.078), while 211-138, 55-164, 211-148, and 23-53 also ranked among the lowest (Table S1). Based on the integrated D values and group assignments under the present experimental conditions, 55-109, YCE07-65, 55-43, 211-172, and 91-74 were identified as priority candidates for repeated drought-tolerance evaluation and multi-environment field validation.

### 2.6. Leave-One-Trait-Out Analysis Supports Associations of Root Growth and Photosynthetic Traits with Integrated Drought Tolerance

**Figure 6:**
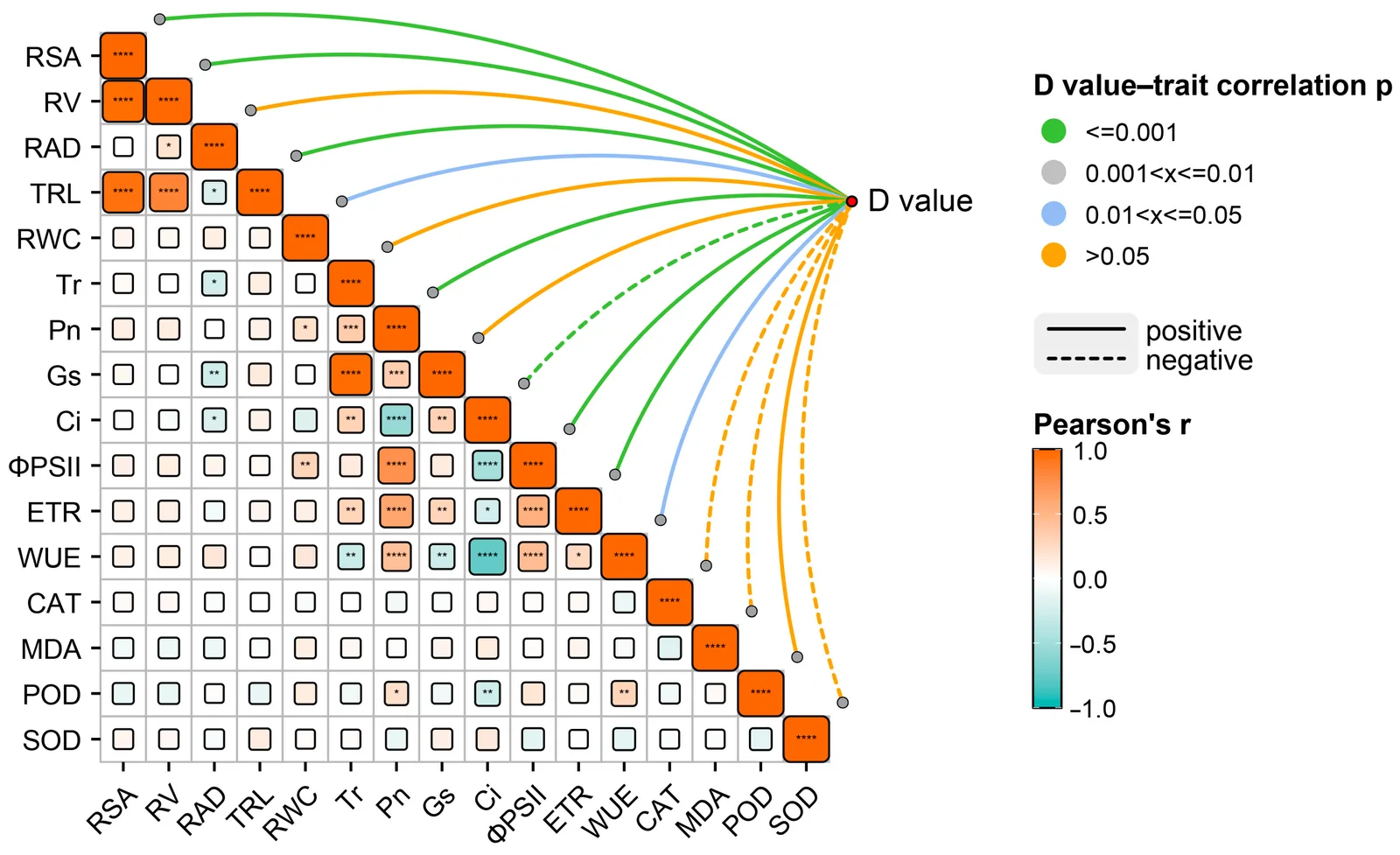
Correlations among 16 drought-response traits and associations of individual traits with integrated drought-tolerance performance. The matrix on the left shows Pearson correlations among the drought-tolerance coefficients of the 16 traits, with color intensity indicating correlation strength and asterisks indicating significance. Connecting lines on the right show trait–D correlations. For the 12 traits included in the integrated D calculation, correlations were calculated using the corresponding leave-one-trait-out D values; for RAD, Tr, Gs, and Ci, which were not included in the D calculation, correlations with the original D value were used. Solid and dashed lines indicate positive and negative correlations, respectively. Line colors indicate significance levels: green, *p* ≤ 0.001; gray, 0.001 < *p* ≤ 0.01; blue, 0.01 < *p* ≤ 0.05; and orange, *p* > 0.05. * *p* < 0.05, ** *p* < 0.01, *** *p* < 0.001, and **** *p* < 0.0001. Detailed correlation coefficients and *p* values are provided in Tables S2–S4. RSA, root surface area; RV, root volume; RAD, root average diameter; TRL, total root length; RWC, relative water content; Tr, transpiration rate; Pn, net photosynthetic rate; Gs, stomatal conductance; Ci, intercellular CO_2_ concentration; ΦPSII, effective quantum yield of PSII photochemistry; ETR, electron transport rate; WUE, water-use efficiency; CAT, catalase activity; MDA, malondialdehyde content; POD, peroxidase activity; SOD, superoxide dismutase activity. The original drought-tolerance coefficient for MDA was used in the correlation analysis, whereas MDA was directionally transformed as a negative indicator when calculating the integrated D value.

Correlation analysis was performed to characterize relationships among the 16 drought-response traits and their associations with integrated drought-tolerance performance (Figure 6). Among the root traits, RSA, RV, and TRL were significantly positively correlated with one another (Tables S2 and S3). To avoid the mathematical dependence arising from direct inclusion of a trait in the integrated D value, associations for the 12 traits included in the D calculation were evaluated using leave-one-trait-out D values. RSA (*r* = 0.533), RV (*r* = 0.519), and TRL (*r* = 0.469) were significantly positively correlated with the leave-one-trait-out D values (*p* < 0.001; Table S4).

Among the photosynthesis-related traits, Pn (*r* = 0.427), ΦPSII (*r* = 0.469), and ETR (*r* = 0.356) were also significantly positively correlated with the leave-one-trait-out D values (*p* < 0.001; Table S4). WUE and RWC showed weaker but significant positive correlations (*r* = 0.237, *p* = 0.0177; and *r* = 0.220, *p* = 0.0276, respectively). In contrast, CAT, POD, SOD, and MDA showed no significant associations with the leave-one-trait-out D values (*p* > 0.05).

RAD, Tr, Gs, and Ci were not included in the integrated D calculation; therefore, their correlations were evaluated directly against the original D value. Ci was significantly negatively correlated with D (*r* = −0.388, *p* < 0.001), whereas RAD, Tr, and Gs showed no significant correlations (*p* > 0.05; Table S4). Most photosynthesis-related traits were positively correlated with one another, whereas Ci was negatively correlated with several of these traits (Tables S2 and S3). This pattern may reflect the balance between stomatal CO_2_ supply and mesophyll CO_2_ assimilation, which can cause Ci to respond differently among genotypes under drought. Overall, root growth and photosynthetic performance showed more consistent associations with integrated drought-tolerance performance than antioxidant enzyme activities or MDA accumulation.

### 2.7. Principal Component Analysis Reveals Multivariate Differentiation Among Drought-Tolerance Groups

**Figure 7:**
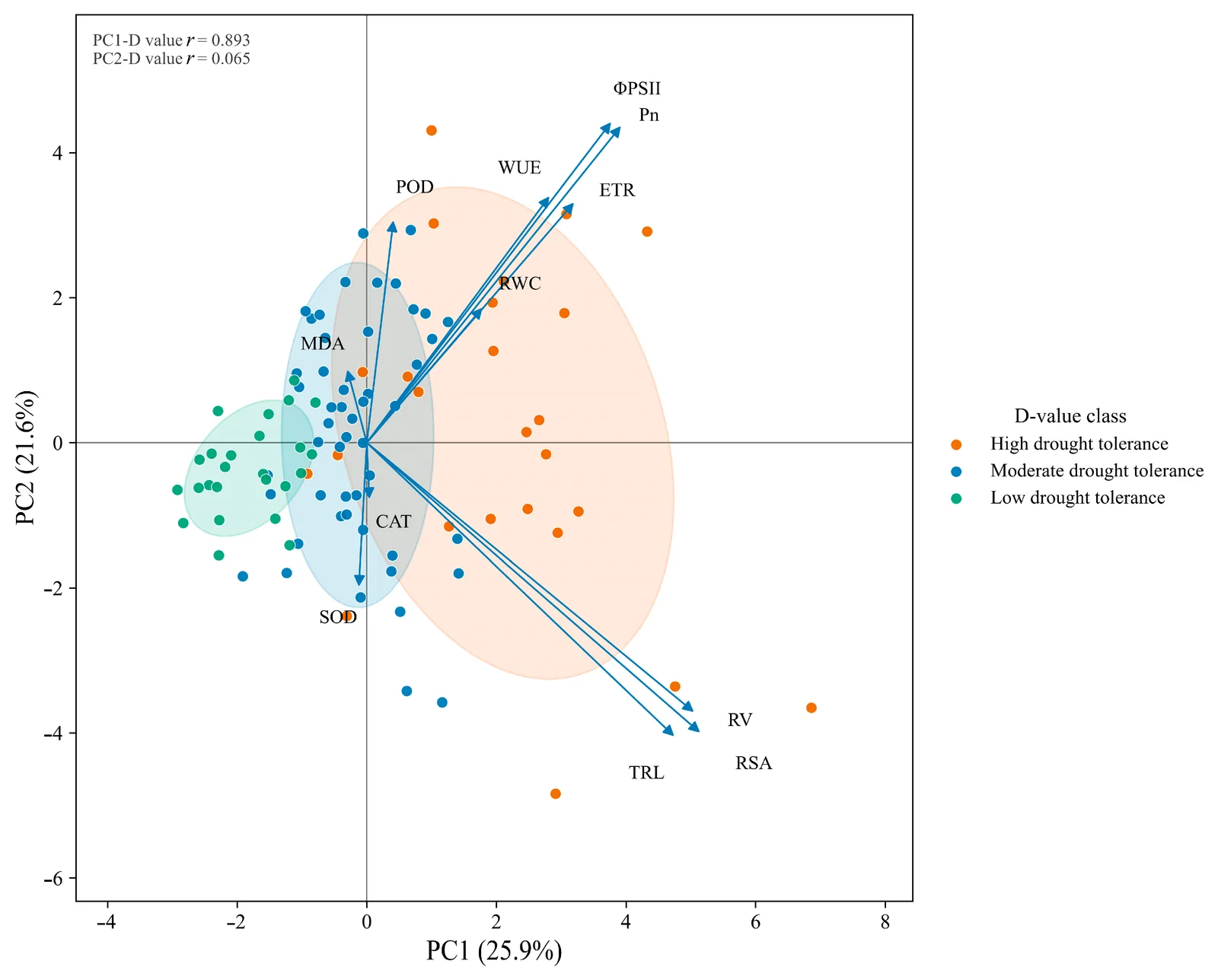
Principal component analysis (PCA) biplot based on 12 drought-tolerance evaluation traits. PC1 and PC2 accounted for 25.9% and 21.6% of the total variance, respectively. Each point represents one genotype. Orange, blue, and green indicate groups with relatively high, moderate, and low drought tolerance within the present germplasm collection, respectively. Semi-transparent ellipses indicate the distribution of each group in the PC1–PC2 space. Blue arrows represent the loading vectors of individual traits on the first two principal components; arrow direction indicates the direction of association with the principal components, and arrow length reflects the magnitude of the loading vector. TRL, total root length; RSA, root surface area; RV, root volume; RWC, relative water content; Pn, net photosynthetic rate; ΦPSII, effective quantum yield of PSII photochemistry; ETR, electron transport rate; WUE, water-use efficiency; CAT, catalase activity; POD, peroxidase activity; SOD, superoxide dismutase activity; MDA, malondialdehyde content.

Principal component analysis was performed using the 12 traits included in the integrated D value evaluation. PC1 and PC2 accounted for 25.85% and 21.56% of the total variance, respectively, explaining 47.41% cumulatively (Figure 7). The three drought-tolerance groups showed a clear gradient along PC1: the low-drought-tolerance group was mainly distributed on the negative side of PC1, the high-drought-tolerance group was more frequently distributed on the positive side, and the moderate-drought-tolerance group was generally positioned between them, although some overlap remained among the groups.

PC1 scores were strongly positively correlated with the integrated D value (*r* = 0.893), whereas the correlation between PC2 scores and the D value was weak (*r* = 0.065), indicating that D value ranking was mainly associated with the multivariate variation represented by PC1. Loading analysis showed that RSA, RV, and TRL had strong positive loadings on PC1. Pn, ΦPSII, ETR, and WUE also loaded positively on PC1. In contrast, CAT, POD, SOD, and MDA showed relatively small loadings on PC1. Overall, the PCA pattern was broadly consistent with the integrated D value classification, with traits related to root growth and photosynthesis contributing strongly to the multivariate gradient represented by PC1.

## 3. Discussion

### 3.1. Coordinated Responses of Root System Architecture and Photosynthetic Function to Drought Stress

Plant adaptation to prolonged water deficit does not depend on a single physiological process but involves the coordination of root water acquisition, maintenance of leaf water status, stomatal regulation, photochemical energy conversion, and reactive oxygen species (ROS) scavenging. Roots are the primary organs for sensing changes in soil water availability and acquiring water, and greater root extension and absorptive surface area can help sustain water uptake under drought. In the present study, drought significantly reduced TRL, RSA, and RV, whereas RAD increased slightly (Figure 1; Table S5), indicating that prolonged water deficit generally restricted root system expansion, while root diameter did not follow the same response pattern as root length, surface area, and volume. Previous studies in sugarcane have similarly shown that drought generally reduces root length, root surface area, root volume, and root biomass, although different genotypes may respond to water deficit by maintaining root length in particular soil layers or altering root distribution, indicating strong genotypic dependence of root responses [31]. Because root depth, root angle, fine-root proportion, and root diameter-class distribution were not measured in this study, the increase in RAD may partly reflect preferential loss of fine roots or structural thickening of the root system and therefore should not be interpreted simply as a favorable drought-adaptive response.

Under drought, reductions in stomatal conductance and transpiration can limit water loss, but prolonged or severe stomatal closure also restricts CO_2_ supply. As drought becomes more severe, PSII photochemistry and electron transport may also be impaired. Considerable genetic variation in stomatal regulation, maintenance of photosynthetic activity, and productivity under water-limited conditions has been reported among sugarcane genotypes, providing an important physiological basis for drought-tolerance screening [10]. In the present study, drought caused large decreases in Pn, Gs, and Tr and significantly reduced ΦPSII and ETR, whereas mean Ci did not differ significantly between treatments but showed a much broader distribution among genotypes under drought (Figure 2; Table S5). Significant genotype × treatment interactions were detected for all gas-exchange and PSII-related traits, providing statistical evidence that the magnitude and/or direction of these responses depended strongly on genotype (Table S5). This pattern does not indicate that Ci was unaffected by drought, but instead suggests that different photosynthetic limitations may have occurred among genotypes. Some genotypes may have reduced Gs and Tr rapidly to limit water loss, thereby restricting CO_2_ diffusion and increasing the contribution of stomatal limitation. In contrast, other genotypes may have maintained or increased Ci despite marked reductions in Pn, ΦPSII, and ETR, suggesting stronger inhibition of mesophyll carbon assimilation or photochemical processes and a greater contribution of non-stomatal limitation. Similar genotypic differences in photosynthetic responses to drought have also been reported in sugarcane [32].

### 3.2. Antioxidant Regulation and Genotypic Variation in Drought Responses

Drought stress not only restricts water acquisition and photosynthetic function but also disrupts cellular redox homeostasis, making the antioxidant response an important physiological component of plant adaptation to drought. In this study, CAT and SOD activities showed significant overall treatment effects, whereas POD showed no significant overall treatment effect. MDA content also increased significantly under drought (Figure 3; Table S5). Significant genotype × treatment interactions were detected for CAT, POD, SOD, and MDA (Table S5), indicating that antioxidant and oxidative-damage responses to drought were strongly genotype-dependent. SOD converts superoxide anions (O_2_^−^) to H_2_O_2_, which must then be further detoxified by antioxidant enzymes such as CAT, POD, and APX. Therefore, mitigation of oxidative stress depends on the coordinated action of multiple antioxidant components rather than on increased activity of a single enzyme. The absence of a significant overall treatment effect for POD, together with its significant genotype × treatment interaction, further indicates that POD responses differed markedly among genotypes and that responses in opposite directions may have partially offset one another at the population level. The marked induction of SOD activity indicates that most genotypes mounted a defensive response to drought-induced oxidative stress, whereas the concurrent accumulation of MDA shows that this response did not completely prevent membrane lipid peroxidation. This pattern suggests that genotypes may differ in ROS-scavenging efficiency and their capacity to maintain redox homeostasis. Studies in grasses such as rice and wheat have likewise shown substantial genotypic variation in antioxidant metabolism, ROS accumulation, and drought adaptation, and that drought tolerance generally depends on coordination among multiple defense processes rather than enhancement of a single antioxidant enzyme [33,34]. Consistent with the significant genotype × treatment interactions, the multitrait heatmap (Figure 4) further showed that, even under the same drought treatment, the progenies differed markedly in both the magnitude and direction of their responses related to root maintenance, photosynthetic function, and antioxidant defense. Such variation within the population may reflect different combinations of water acquisition, photosynthetic maintenance, and antioxidant capacity among genetic backgrounds. Therefore, a high value for any single root, photosynthetic, or antioxidant trait cannot by itself be equated with greater overall drought tolerance, supporting the use of multiple traits with biologically interpretable response directions for integrated drought-tolerance evaluation.

### 3.3. Integrated Multitrait Evaluation Identifies Phenotypic Features Associated with Drought Tolerance in Sugarcane

Drought tolerance is a complex quantitative trait determined by multiple morphological, physiological, and biochemical traits. Because these traits differ in their sensitivity to water deficit, direction of response, and environmental stability, a single trait is unlikely to fully capture overall drought tolerance. Integrated evaluation based on multiple traits has therefore been widely used for drought-tolerance assessment in cereal crops. Chen et al. integrated drought-tolerance coefficients for multiple morphological, physiological, and yield-related traits in winter wheat and used membership function analysis to evaluate genotypic variation in drought tolerance [14]. Similar approaches have also been applied in sorghum and sugarcane [17,35,36]. These studies indicate that multitrait evaluation requires not only the integration of different aspects of drought response but also the selection of traits with relatively consistent response directions and clear biological interpretation. Accordingly, 12 of the 16 measured traits were selected for calculation of the integrated D value in this study, whereas Ci, Gs, Tr, and RAD were excluded. The responses of Gs, Tr, and Ci are strongly influenced by environmental conditions and genotype. Stomatal closure can reduce transpirational water loss but also restrict CO_2_ diffusion, while Ci may increase or decrease depending on the relative contributions of stomatal and non-stomatal limitations [37,38]. RAD is also affected by the composition of different root diameter classes and adjustments in root system architecture, making it difficult to assign a consistent unidirectional relationship with drought tolerance [39].

The leave-one-trait-out analysis provided a more conservative assessment of the associations between individual traits and integrated drought-tolerance performance by removing the direct mathematical contribution of each tested trait from the D value. RSA, RV, TRL, Pn, ΦPSII, and ETR remained significantly positively associated with the corresponding leave-one-trait-out D values, while WUE and RWC showed weaker but still significant associations. In contrast, CAT, POD, SOD, and MDA showed no significant associations with the leave-one-trait-out D values (Figure 6; Table S4). These results indicate that the relationships of root growth and photosynthetic performance with overall drought-tolerance ranking were not solely caused by the direct inclusion of these traits in the D calculation. This does not imply that antioxidant metabolism or lipid peroxidation is unimportant in drought response; rather, variation in these traits among genotypes was not consistently aligned with the overall drought-tolerance ranking in the present population. PCA showed a broadly similar multivariate pattern: PC1 explained 25.85% of the total variance and was strongly correlated with the integrated D value (*r* = 0.893), while root- and photosynthesis-related traits loaded predominantly on the positive side of PC1 (Figure 7). Together, these results suggest that maintenance of root growth and photosynthetic function represented more consistent phenotypic features associated with higher overall drought-tolerance performance under the present experimental conditions. However, these associations should be interpreted cautiously because the integrated D value and PCA were derived from the same set of traits. Consequently, the agreement between the PCA pattern and D value ranking primarily reflects the internal consistency of the composite evaluation framework rather than an independent validation of the drought-tolerance classification or of the contribution of individual traits. The selected traits and high-ranking genotypes therefore require confirmation in independent drought experiments and across multiple years, water regimes, and field environments before they can be considered stable criteria or germplasm resources for drought-tolerance breeding.

One limitation of the present study is that shoot fresh weight, shoot dry weight, and total plant biomass were not measured. The present evaluation focused primarily on root system architecture, leaf water status, photosynthetic performance, and antioxidant responses to drought. Consequently, the integrated evaluation does not directly capture drought-induced changes in aboveground growth or whole-plant biomass accumulation. Future studies should incorporate shoot biomass, total biomass, and root-to-shoot allocation to provide a more comprehensive assessment of drought tolerance.

### 3.4. Potential Contribution of T. arundinaceum-Derived Introgression to Drought-Tolerance Breeding in Sugarcane

Crop wild relatives are important resources for broadening the genetic diversity of cultivated crops and identifying favorable allelic variation for stress tolerance. Studies in cereals such as wheat and barley have shown that the incorporation of genetic variation from wild germplasm through wide hybridization or introgression breeding can increase phenotypic variation for drought tolerance and generate progeny with improved performance under water deficit [40,41]. For modern sugarcane, which has a highly complex genetic background, the use of wild relatives is likewise an important approach for broadening the genetic basis of stress tolerance. *T. arundinaceum* has strong root growth, ratooning ability, and adaptation to adverse environments and has long been used as a wild genetic resource in intergeneric sugarcane breeding. Cytogenetic studies have shown that *T. arundinaceum*-derived chromosomes can be introgressed into the sugarcane genetic background through intergeneric hybridization followed by successive backcrossing, with varying patterns of chromosome transmission, recombination, and translocation in subsequent progenies [25,26]. Introgression of *T. arundinaceum*-derived chromosomes has also been associated with variation in agronomic traits such as biomass, stalk diameter, and single-stalk weight [21].

Recent work further showed that *T. arundinaceum* has relatively high intrinsic water-use efficiency and strong root-forming ability, while sugarcane × *T. arundinaceum* intergeneric hybrids can combine, to some extent, physiological characteristics derived from both parental backgrounds [28]. These findings support the potential value of *T. arundinaceum* germplasm for broadening phenotypic variation related to water use and drought adaptation in sugarcane. In the present study, several genotypes carrying *T. arundinaceum*-derived genetic material showed high integrated D values, and some ranked higher than the drought-tolerant control XTT22 (Figure 5; Table 2) [3,42]. Marker-based chromosome assignments further confirmed the presence of *T. arundinaceum*-derived chromosomes in all five of the highest-ranking genotypes identified in this study (55-109, YCE07-65, 55-43, 211-172, and 91-74; Table S6). These high-ranking materials provide useful germplasm for further investigating the potential contribution of *T. arundinaceum*-derived genetic variation to drought adaptation in sugarcane. However, the coexistence of *T. arundinaceum*-derived chromosomes and high integrated D values does not by itself establish a causal relationship between individual introgressed chromosomes and drought tolerance. In addition, interpretation is complicated by the pedigree structure of the experimental population. The five progeny populations were derived from different parental combinations, and the backcross generation was confounded with family background: populations 23, 91, and 213 were BC_4_F_1_, whereas populations 55 and 211 were BC_5_F_1_ (Table 3). Consequently, differences in drought response between BC_4_F_1_ and BC_5_F_1_ materials cannot be attributed to backcross generation per se, because they may also reflect parental genetic effects, relatedness or shared ancestry among materials, segregation within families, and interactions between sugarcane- and *T. arundinaceum*-derived loci. The present experimental design therefore does not allow the effect of backcross generation to be estimated independently from family and parental background. Further analyses combining chromosome-specific molecular markers, genomic in situ hybridization (GISH), whole-genome resequencing, and chromosome- or segment-level association analysis will be required to determine whether particular *T. arundinaceum*-derived genomic regions contribute reproducibly to drought tolerance. Such analyses would provide a stronger basis for identifying favorable introgressed regions and evaluating their potential use in broadening the genetic basis of drought-tolerance breeding in modern sugarcane.

## 4. Materials and Methods

### 4.1. Plant Materials

Plant materials used in this study comprised 100 genotypes, including 93 advanced backcross progenies and seven additional parental or reference genotypes. The 93 progenies were derived from five BC_4_F_1_ or BC_5_F_1_ cross combinations, designated 23, 55, 91, 211, and 213 (Table 3). The seven additional genotypes were YCE06-61, YCE07-65, YCE05-164, YCE07-71, YCE05-64, XTT22, and YT93-159 (Table 4). The five YCE genotypes were included as parental materials associated with the advanced backcross populations and carry *T. arundinaceum*-derived chromosomes, thereby providing relevant genetic references for comparison with their progenies. XTT22 was included as a previously characterized drought-tolerant reference genotype [42], whereas YT93-159 was included as a drought-sensitive reference genotype [43]. The *T. arundinaceum*-derived chromosome composition of selected advanced backcross materials was determined using *T. arundinaceum* chromosome-specific molecular markers developed by our research group. These markers enabled identification of the corresponding *T. arundinaceum*-derived chromosome types present in individual genotypes. Marker-based chromosome assignments, including those of all five highest-ranking genotypes identified in the present drought-tolerance evaluation, are provided in Table S6. All materials were grown and propagated in Xiamao Town, Shaxian District, Sanming, Fujian Province, China.

### 4.2. Plant Cultivation and Drought Treatment

For each genotype, 6–10 single-bud setts were excised from mature stalks collected from multiple clonal stalks of the same genotype and pre-germinated under uniform conditions. When the emerging shoots reached approximately 2–3 cm in length, six uniformly growing seedlings were selected and transplanted individually into pots. Each pot contained one plant and was regarded as an independent experimental unit.

The experiment was conducted in September 2024 in a greenhouse at the Jinshan Campus of Fujian Agriculture and Forestry University. During this period, the local mean daily maximum and minimum air temperatures were 30 °C and 25 °C, respectively, and the mean daily sunshine duration was 8.83 h. Black plastic pots were used as cultivation containers, with a bottom diameter of 32 cm, a top diameter of 35 cm, and a height of 28 cm. Each pot was filled with 17 kg of yellow soil.

For each genotype, three independently grown plants were assigned to the well-watered control and three to the drought treatment, resulting in three biological replicates per genotype × treatment combination and 600 pots in total. The control and drought treatments were arranged in separate greenhouse areas to facilitate independent irrigation management and to prevent accidental water movement from the control pots to the drought-treated pots. Within each treatment area, the plants were arranged in three blocks, with one pot per genotype included in each block.

Before the initiation of drought treatment, all plants received the same irrigation and fertilization management. When the seedlings reached the seven-leaf stage, the drought treatment was initiated. Before treatment, all pots were thoroughly irrigated to ensure relatively uniform soil moisture conditions. Soil volumetric water content (VWC) was monitored approximately every 3 d using a TZS-1K-G soil moisture meter (Zhejiang Top Cloud-Agri Technology Co., Ltd., Hangzhou, China), with the probe inserted to a soil depth of approximately 14 cm. The values displayed by the instrument were recorded directly as %VWC. During the treatment period, soil VWC in the well-watered control was maintained at approximately 70–80%, whereas irrigation was withheld from the drought-treatment group, allowing soil VWC to decline progressively. After 20 d of water withholding, soil VWC in the drought-treated pots had decreased to approximately 25%, accompanied by pronounced leaf rolling, wilting, and yellowing.

At the end of the treatment, photosynthetic parameters were measured first. For each genotype and treatment, one plant was sampled from each of three independent pots. Each pot was regarded as one biological replicate, resulting in three biological replicates per treatment. The second fully expanded leaf from the top was collected and divided into subsamples. One portion was used immediately for relative water content determination, whereas the remaining portion was immediately frozen in liquid nitrogen and stored at −80 °C for subsequent physiological and biochemical analyses. Root systems were carefully excavated, washed with clean water, and immediately scanned for root morphological analysis. After scanning, the root samples were placed in sealable plastic bags and stored at −80 °C.

### 4.3. Trait Measurements

#### 4.3.1. Measurement of Gas Exchange and Photosynthetic Parameters

At the end of the drought treatment, gas exchange and photosynthetic parameters were measured in both control and drought-treated plants between 08:00 and 11:00 using an LI-6800 portable photosynthesis and fluorescence system (LI-COR Biosciences, Lincoln, NE, USA). The measured parameters included net photosynthetic rate (Pn, μmol CO_2_ m^−2^ s^−1^), stomatal conductance (Gs, mol H_2_O m^−2^ s^−1^), intercellular CO_2_ concentration (Ci, μmol mol^−1^), effective quantum yield of PSII photochemistry (ΦPSII), electron transport rate (ETR), and transpiration rate (Tr, mmol H_2_O m^−2^ s^−1^). Instantaneous water-use efficiency (WUE) was calculated as the ratio of Pn to Tr.

#### 4.3.2. Measurement of Root Traits and Leaf Relative Water Content

The root systems were separated from the aboveground parts of the plants, carefully washed, and scanned using an EPSON Perfection Photo root scanner. Root traits were then analyzed using WinRHIZO (Pro Software Version 2021a, Regent Instruments Inc., Quebec, QC, Canada) software.

For determination of leaf relative water content (RWC), the second fully expanded leaf from the top of the plant was collected and weighed immediately to obtain the fresh weight (*W_t_*). The samples were then immersed in distilled water for 8 h to achieve full hydration. After excess surface water was removed with filter paper, the leaves were weighed to obtain the turgid weight (*W_s_*). The samples were subsequently placed in paper bags and dried to constant weight in an oven at 90 °C to determine the dry weight (*W_d_*). Leaf RWC was calculated according to Equation (1).

(1) $$\mathrm{Rwc}(\%)=\frac{w_{t}-w_{d}}{w_{S}-w_{d}}\times 100$$

#### 4.3.3. Physiological and Biochemical Measurements

The activities of SOD, POD, and CAT, and the content of MDA were determined using commercial assay kits from Beijing Solarbio Science & Technology Co., Ltd. (Beijing, China). The corresponding catalog numbers were BC0175, BC0095, BC0205, and BC0025, respectively. All measurements were performed according to the manufacturer’s instructions.

### 4.4. Integrated Evaluation of Drought Tolerance

Drought tolerance of the 100 sugarcane genotypes was evaluated using a membership-function-based integrated D value approach adapted from established multitrait drought-tolerance evaluation frameworks rather than newly developed in the present study [14,17,18,36]. The general framework involves calculating drought-tolerance coefficients, transforming individual traits into membership function values, and integrating information across multiple traits into a composite drought-tolerance score. In the present implementation, the relative contribution of each selected trait was further accounted for using weights derived from the coefficients of variation of its membership function values.

The drought-tolerance coefficient (*X_ij_*) was calculated as:

(2) $$\left(X_{ij}\right)=\frac{D_{ij}}{{CK}_{ij}}$$

where *X_ij_* is the drought-tolerance coefficient of the *jth* trait for the *ith* genotype; *D_ij_* is the measured value of the *jth* trait for the *ith* genotype under drought treatment; and *CK_ij_* is the corresponding value under the well-watered control condition.

For positive indicators, the membership function value, *μ*(*X_ij_*), was calculated as:

(3) $$\mu \left(X_{ij}\right)=\frac{X_{ij}-X_{\mathrm{jmin}}}{X_{\mathrm{jmax}}-X_{\mathrm{jmin}}}$$

For negative indicators, the membership function value was calculated as:

(4) $$\mu \left(X_{ij}\right)=\frac{X_{\mathrm{jmax}}-X_{ij}}{X_{\mathrm{jmax}}-X_{\mathrm{jmin}}}$$

where *μ*(*X_ij_*) is the membership function value of the *jth* trait for the *ith* genotype; *X_ij_* is the drought-tolerance coefficient of the *jth* trait for the *ith* genotype; and *X_jmax_* and *X_jmin_* are the maximum and minimum drought-tolerance coefficients, respectively, for the *jth* trait among all tested genotypes. A membership function value closer to 1 indicates better drought-tolerance performance for that trait, whereas a value closer to 0 indicates poorer performance.

The coefficient of variation for each trait was calculated as:

(5) $$CV_{j}=\frac{\sqrt{\frac{1}{n}\sum_{i=1}^{n}{\left(\mu \left(X_{ij}\right)-{\bar{X}}_{j}\right)}^{2}}}{\bar{x_{j}}}$$

where *CV_j_* is the coefficient of variation of the membership function values for the *jth* trait; *n* is the total number of tested genotypes; *μ*(*X_ij_*) is the membership function value of the *jth* trait for the *ith* genotype; and *μ*(*X_j_*) is the mean membership function value of the *jth* trait across all genotypes.

The weight of each trait was calculated as:

(6) $$W_{j}=\frac{CV_{j}}{\sum_{k=1}^{m}CV_{k}}$$

where *W_j_* is the weight of the *jth* trait, *CV_j_* is the coefficient of variation of the *jth* trait, and *m* is the total number of traits included in the integrated evaluation; *k* is the summation index over all traits.

The integrated D value was calculated as:

(7) $$D_{i}=\sum_{j=1}^{m}\left(\mu \left(x_{ij}\right)\times W_{j}\right)$$

where *D_i_* is the integrated drought-tolerance value of the *ith* sugarcane genotype. A higher D value indicates better overall drought-tolerance performance under the experimental conditions.

### 4.5. Statistical Analysis

#### 4.5.1. Statistical Testing and Data Visualization

Data organization and preliminary calculations were performed in Microsoft Excel. For graphical summaries and calculation of drought-tolerance coefficients, the mean of the three biological replicates was calculated for each genotype under each treatment. For statistical inference on each of the 16 measured traits, the original pot-level observations were analyzed using a two-factor linear model including genotype, treatment, and genotype × treatment interaction as fixed effects. The three independent pots for each genotype × treatment combination were retained as biological replicates. The genotype effect was used to assess differences among the 100 genotypes, the treatment effect represented the average effect of drought across the tested genotypes, and the genotype × treatment interaction was used to determine whether the magnitude and/or direction of drought responses differed among genotypes. The two-factor linear models were fitted using Python 3.11. Differences were considered statistically significant at *p* < 0.05, with additional significance levels reported at *p* < 0.01, *p* < 0.001, and *p* < 0.0001. Boxplots were generated using GraphPad Prism 11.

#### 4.5.2. Heatmap Analysis of Drought Responses

The drought-tolerance coefficient (D/CK) was first calculated for each trait of each genotype, where D and CK represent the trait values under drought and well-watered control conditions, respectively. The D/CK values were then log_2_-transformed. To facilitate comparison of response magnitudes among traits, the log_2_ (D/CK) values for each trait were normalized by dividing each value by the maximum absolute value of that trait across all genotypes. The normalized data were used to generate a drought-response heatmap using TBtools v2.485.

#### 4.5.3. Hierarchical Clustering and Drought-Tolerance Classification

Euclidean distances among genotypes were calculated based on the integrated D values, followed by hierarchical clustering using the Ward.D2 linkage method. The resulting hierarchical clustering dendrogram showed three major clusters, which were interpreted as groups with relatively high, moderate, and low drought tolerance within the present germplasm collection. To obtain numerical thresholds for classification based on the integrated D value, genotypes were ranked in descending order of D value, and the boundary genotypes between adjacent clusters were identified. The midpoint between the D values of the two genotypes located on either side of each cluster boundary was used as the classification threshold. This procedure yielded dataset-specific thresholds of 0.297 between the high- and moderate-drought-tolerance groups and 0.196 between the moderate- and low-drought-tolerance groups. Hierarchical clustering, D value ranking, and visualization of drought-tolerance groups were performed in Python 3.11.

#### 4.5.4. Correlation Analysis and Principal Component Analysis

Pearson correlation analysis was used to assess relationships among the drought-tolerance coefficients of the 16 traits. For the 12 traits included in the integrated D value calculation, leave-one-trait-out correlations were calculated by excluding each trait in turn, recalculating D using the remaining 11 traits with renormalized weights, and correlating the excluded trait with the corresponding recalculated D value. For RAD, Tr, Gs, and Ci, which were not included in the D calculation, correlations with the original D value were retained. For MDA, the original D/CK ratio was used for correlation analysis. Principal component analysis (PCA) was performed using the membership function values of the 12 traits included in the integrated D value calculation. Before PCA, the membership function values were Z-score standardized to place the traits on a comparable scale. Scores and loadings of the first two principal components were used to examine the distribution of drought-tolerance groups in multivariate trait space and the contributions of individual traits to the major axes of variation. PCA and its visualization were performed in Python 3.11.

## 5. Conclusions

This study evaluated drought tolerance in advanced sugarcane × *T. arundinaceum* backcross progenies and related materials using multiple morphological, physiological, and biochemical traits. Twenty days of drought markedly restricted root system expansion, leaf water status, gas exchange, and PSII photochemical performance while inducing antioxidant responses and lipid peroxidation. Significant genotype × treatment interactions across all 16 measured traits further demonstrated substantial genotype-dependent variation in drought responses. Based on 12 traits with relatively consistent response directions, the integrated D value evaluation classified the 100 genotypes into groups with relatively high, moderate, and low drought tolerance within the present germplasm collection. Genotypes 55-109, YCE07-65, 55-43, 211-172, and 91-74 ranked highest and represent priority candidates for further drought-tolerance evaluation and field validation. Correlation analysis and PCA consistently associated higher D values with maintenance of root growth and photosynthetic function, particularly RSA, RV, TRL, Pn, ΦPSII, and ETR. The advanced backcross populations carrying *T. arundinaceum*-derived genetic material also showed substantial phenotypic variation for drought tolerance. However, the superior performance of individual genotypes cannot yet be attributed to specific *T. arundinaceum* chromosomes or introgressed segments. Multi-year and multi-environment field evaluation, together with characterization of introgressed genomic regions, will be required to confirm the stability and genetic basis of the selected drought-tolerant materials.

## Figures and Tables

**Table 1:** Statistical characteristics of drought-tolerance coefficients for 16 drought-response traits in 100 sugarcane genotypes.

Trait	Drought-Tolerance Coefficient (Mean ± SD)	Range
TRL	0.7195 ± 0.5053	0.1096–2.7346
RSA	0.7395 ± 0.5307	0.1061–3.1061
RAD	1.0695 ± 0.2329	0.5530–2.1370
RV	0.7905 ± 0.6133	0.1216–3.9442
RWC	0.8550 ± 0.1554	0.2759–1.2600
Tr	0.2977 ± 0.2381	0.0285–1.5770
Pn	0.2427 ± 0.2172	0.0045–1.3014
Gs	0.2722 ± 0.2526	0.0278–1.6677
Ci	1.0059 ± 0.4230	0.0893–2.4468
ΦPSII	0.5769 ± 0.2056	0.0697–1.2143
ETR	0.4440 ± 0.2914	0.0005–1.2142
WUE	1.0617 ± 1.0859	0.0092–5.5271
CAT	2.2983 ± 3.1955	0.0930–23.4984
POD	1.1448 ± 0.5613	0.3660–3.1017
SOD	4.0437 ± 4.9177	0.0746–23.7081
MDA	1.5046 ± 0.3586	1.0463–2.7897

**Table 2:** Membership function values, integrated D values, and drought-tolerance rankings of the top 15 genotypes.

Genotype	Membership Function Value												D Value	Rank
	TRL	RSA	RV	CAT	POD	SOD	MDA	Pn	ΦPSII	ETR	WUE	RWC		
55-109	0.870	1.000	1.000	0.110	0.241	0.415	0.499	0.360	0.571	0.596	0.340	0.600	0.516	1
YCE07-65	0.383	0.358	0.292	0.007	0.526	0.350	0.966	0.367	0.647	0.667	0.818	0.671	0.423	2
55-43	0.374	0.383	0.343	0.014	0.483	0.011	0.780	1.000	0.839	0.849	0.247	0.555	0.394	3
211-172	0.220	0.233	0.214	0.004	0.163	0.268	0.905	0.379	1.000	1.000	0.694	0.732	0.377	4
91-74	0.634	0.280	0.134	0.002	0.119	0.997	0.941	0.335	0.473	0.504	0.065	0.634	0.370	5
55-146	0.696	0.529	0.349	0.039	0.398	0.350	0.562	0.297	0.546	0.572	0.157	0.858	0.370	6
213-7	1.000	0.841	0.601	0.113	0.230	0.029	0.583	0.268	0.568	0.026	0.120	0.891	0.363	7
91-81	0.198	0.181	0.143	1.000	0.538	0.092	0.773	0.060	0.413	0.446	0.142	0.596	0.360	8
55-74	0.527	0.540	0.484	0.070	0.283	0.021	0.321	0.118	0.299	0.339	1.000	0.507	0.353	9
213-15	0.630	0.754	0.777	0.462	0.118	0.140	0.406	0.002	0.319	0.358	0.001	0.370	0.353	10
55-80	0.211	0.259	0.268	0.180	0.105	0.297	0.839	0.434	0.644	0.665	0.397	0.733	0.343	11
55-68	0.071	0.070	0.057	0.015	1.000	0.009	0.974	0.354	0.681	0.699	0.834	0.516	0.341	12
91-79	0.126	0.093	0.057	0.000	0.668	0.440	0.656	0.480	0.728	0.743	0.313	0.668	0.332	13
23-54	0.490	0.423	0.323	0.059	0.278	0.003	0.914	0.346	0.601	0.624	0.426	0.489	0.329	14
XTT22	0.253	0.218	0.163	0.020	0.272	0.308	0.829	0.646	0.713	0.729	0.142	0.851	0.326	15
Trait weight	0.087	0.088	0.096	0.152	0.076	0.130	0.029	0.096	0.042	0.069	0.108	0.028		

**Table 3:** Cross combinations, generations, and parental information of the sugarcane × *T. arundinaceum* progeny populations used in this study.

Cross Combination	Generation	Female Parent (♀)	Male Parent (♂)	No. of Genotypes
23	BC_4_F_1_	LC03-1137	YCE06-61	6
55	BC_5_F_1_	YCE07-65	XTT22	36
91	BC_4_F_1_	FN07-12	YCE05-164	26
211	BC_5_F_1_	YT00-236	YCE07-71	15
213	BC_4_F_1_	FN023924	YCE05-64	10

**Table 4:** Identity and experimental roles of the additional parental and drought-reference genotypes included in this study.

Role in This Study	Genotype
Parental reference	YCE06-61
Parental reference	YCE07-65
Parental reference	YCE05-164
Parental reference	YCE07-71
Parental reference	YCE05-64
Drought-tolerant reference	XTT22
Drought-sensitive reference	YT93-159

## Data Availability

Data are contained within the article and Supplementary Materials.

## Supplementary Materials

The following supporting information can be downloaded at https://www.mdpi.com/article/10.3390/plants15182813/s1, Table S1: Membership Function Values, Integrated D Values, and Drought-Tolerance Rankings of the 100 Genotypes; Table S2: Pearson Correlation Coefficients Among the Drought-Tolerance Coefficients of the 16 Drought-Response Traits; Table S3: *p* Values Corresponding to the Pearson Correlations Among the Drought-Tolerance Coefficients of the 16 Drought-Response Traits; Table S4: Pearson Correlations Between Individual Trait Drought-Tolerance Coefficients and Integrated Drought-Tolerance Performance Using the Leave-One-Trait-Out Approach; Table S5: Effects of Genotype, Drought Treatment, and Their Interaction on the 16 Drought-Response Traits; Table S6: Marker-Based Detection of *Tripidium arundinaceum*-Derived Chromosome Types in Selected Advanced Backcross and Parental/Reference Genotypes.
