# Supplementary material for: Multitrait Evaluation of Drought Tolerance at the Seedling Stage in Advanced Backcross Germplasm Derived from Sugarcane × Tripidium arundinaceum
Source: Plants (Basel). 2026 Sep 14;15(18):2813. doi: 10.3390/plants15182813 (PMC13609959; doi:10.3390/plants15182813)
Supplement: Supplementary file 1 [file plants-15-02813-s001.zip › Supplementary_Tables_S1.pdf]

Table S1. Membership Function Values, Integrated D Values, and Drought-Tolerance Rankings of the 100 Genotypes.

Genotype	Membership function value												D-value	Rank
	TRL	RSA	RV	CAT	POD	SOD	MDA	Pn	ΦPSII	ETR	WUE	RWC		
55-109	0.870	1.000	1.000	0.110	0.241	0.415	0.499	0.360	0.571	0.596	0.340	0.600	0.516	1
YCE07-65	0.383	0.358	0.292	0.007	0.526	0.350	0.966	0.367	0.647	0.667	0.818	0.671	0.423	2
55-43	0.374	0.383	0.343	0.014	0.483	0.011	0.780	1.000	0.839	0.849	0.247	0.555	0.394	3
211-172	0.220	0.233	0.214	0.004	0.163	0.268	0.905	0.379	1.000	1.000	0.694	0.732	0.378	4
91-74	0.634	0.280	0.134	0.002	0.119	0.997	0.941	0.335	0.473	0.504	0.065	0.634	0.371	5
55-146	0.696	0.529	0.349	0.039	0.398	0.350	0.562	0.297	0.546	0.572	0.157	0.858	0.370	6
213-7	1.000	0.841	0.601	0.113	0.230	0.029	0.583	0.268	0.568	0.026	0.120	0.891	0.363	7
91-81	0.198	0.181	0.143	1.000	0.538	0.092	0.773	0.060	0.413	0.446	0.142	0.596	0.360	8
55-74	0.527	0.540	0.484	0.070	0.283	0.021	0.321	0.118	0.299	0.339	1.000	0.507	0.354	9
213-15	0.630	0.754	0.777	0.462	0.118	0.140	0.406	0.002	0.319	0.358	0.001	0.370	0.353	10
55-80	0.211	0.259	0.268	0.180	0.105	0.297	0.839	0.434	0.644	0.665	0.397	0.733	0.343	11
55-68	0.071	0.070	0.057	0.015	1.000	0.009	0.974	0.354	0.681	0.699	0.834	0.516	0.341	12
91-79	0.126	0.093	0.057	0.000	0.668	0.440	0.656	0.480	0.728	0.743	0.313	0.668	0.332	13
23-54	0.490	0.423	0.323	0.059	0.278	0.003	0.914	0.346	0.601	0.624	0.426	0.489	0.329	14
XTT22	0.253	0.218	0.163	0.020	0.272	0.308	0.829	0.646	0.713	0.729	0.142	0.851	0.326	15
55-142	0.395	0.287	0.183	0.163	0.148	1.000	0.656	0.043	0.302	0.341	0.046	0.530	0.322	16
91-78	0.219	0.225	0.203	0.242	0.163	0.066	0.884	0.502	0.722	0.738	0.218	0.807	0.317	17
211-243	0.413	0.450	0.434	0.014	0.126	0.034	0.891	0.357	0.640	0.661	0.220	0.533	0.305	18
91-88	0.477	0.488	0.442	0.031	0.191	0.067	0.967	0.314	0.454	0.485	0.175	0.696	0.304	19
23-56	0.301	0.217	0.132	0.338	0.519	0.000	0.851	0.238	0.563	0.588	0.114	0.623	0.290	20
211-101	0.455	0.446	0.393	0.021	0.283	0.097	0.702	0.277	0.387	0.422	0.241	0.562	0.288	21
23-57	0.414	0.415	0.369	0.040	0.276	0.055	0.733	0.331	0.942	0.040	0.253	0.640	0.283	22
55-163	0.286	0.255	0.203	0.036	0.538	0.030	0.650	0.143	0.306	0.346	0.668	0.766	0.280	23
55-127	0.156	0.175	0.174	0.053	0.577	0.630	0.928	0.101	0.330	0.369	0.092	0.441	0.278	24
55-98	0.110	0.120	0.113	0.129	0.035	0.336	1.000	0.178	0.462	0.493	0.583	0.612	0.277	25
91-65	0.163	0.151	0.119	0.051	0.505	0.634	0.898	0.097	0.374	0.410	0.088	0.693	0.276	26

55-78	0.105	0.109	0.094	0.278	0.127	0.178	0.021	0.419	0.744	0.759	0.255	0.592	0.271	27
91-86	0.482	0.485	0.429	0.017	0.217	0.537	0.690	0.000	0.259	0.000	0.023	0.635	0.266	28
213-1	0.213	0.184	0.137	0.276	0.111	0.014	0.711	0.292	0.610	0.632	0.244	0.669	0.263	29
23-27	0.205	0.245	0.256	0.015	0.720	0.007	0.896	0.159	0.766	0.389	0.175	0.718	0.261	30
55-147	0.222	0.141	0.074	0.150	0.283	0.027	0.841	0.345	0.636	0.657	0.188	0.760	0.258	31
55-168	0.203	0.210	0.204	0.031	0.457	0.017	0.550	0.327	0.831	0.420	0.267	0.578	0.254	32
55-103	0.284	0.204	0.132	0.319	0.229	0.547	0.769	0.035	0.374	0.005	0.070	0.425	0.254	33
91-38	0.132	0.148	0.144	0.006	0.654	0.094	0.878	0.305	0.444	0.476	0.286	0.520	0.253	34
55-160	0.113	0.096	0.070	0.021	0.924	0.061	0.975	0.310	0.402	0.437	0.171	0.778	0.252	35
213-12	0.094	0.109	0.104	0.198	0.093	0.449	0.678	0.207	0.473	0.503	0.168	0.568	0.251	36
211-58	0.089	0.064	0.036	0.001	0.224	0.068	0.793	0.527	0.702	0.719	0.381	0.503	0.251	37
55-71	0.023	0.045	0.067	0.056	0.695	0.018	0.694	0.319	0.621	0.052	0.658	0.801	0.250	38
55-47	0.108	0.119	0.110	0.174	0.492	0.001	0.738	0.357	0.701	0.030	0.371	1.000	0.250	39
55-73	0.459	0.437	0.370	0.007	0.238	0.006	0.940	0.201	0.360	0.397	0.089	0.597	0.249	40
91-87	0.468	0.447	0.385	0.111	0.045	0.046	0.930	0.003	0.509	0.537	0.013	0.619	0.248	41
213-20	0.269	0.350	0.397	0.325	0.140	0.039	0.612	0.142	0.456	0.016	0.267	0.281	0.246	42
55-83	0.196	0.176	0.136	0.243	0.231	0.255	0.885	0.132	0.412	0.446	0.040	0.744	0.245	43
91-55	0.051	0.023	0.002	0.326	0.240	0.060	0.828	0.319	0.555	0.581	0.238	0.432	0.238	44
55-95	0.205	0.169	0.115	0.409	0.004	0.231	0.740	0.073	0.365	0.401	0.087	0.725	0.237	45
55-144	0.322	0.342	0.321	0.149	0.146	0.281	0.873	0.046	0.214	0.259	0.031	0.640	0.237	46
91-20	0.775	0.524	0.319	0.074	0.037	0.098	0.718	0.009	0.273	0.314	0.005	0.366	0.237	47
YCE07-71	0.465	0.354	0.226	0.026	0.097	0.158	0.898	0.103	0.346	0.014	0.389	0.530	0.234	48
91-42	0.231	0.168	0.103	0.203	0.108	0.129	0.853	0.277	0.378	0.414	0.207	0.481	0.232	49
55-172	0.078	0.097	0.101	0.011	0.568	0.017	0.645	0.374	0.523	0.550	0.233	0.702	0.232	50
211-144	0.434	0.252	0.130	0.003	0.183	0.317	0.543	0.118	0.333	0.372	0.193	0.445	0.228	51
55-140	0.071	0.092	0.102	0.063	0.458	0.274	0.963	0.223	0.445	0.476	0.138	0.273	0.227	52
55-166	0.240	0.198	0.140	0.012	0.449	0.039	0.881	0.187	0.407	0.441	0.238	0.544	0.225	53
91-67	0.176	0.194	0.191	0.017	0.147	0.622	0.842	0.005	0.283	0.324	0.012	0.663	0.225	54
91-6	0.534	0.433	0.311	0.006	0.389	0.063	0.689	0.029	0.246	0.289	0.026	0.450	0.221	55
55-174	0.212	0.212	0.185	0.041	0.183	0.052	0.761	0.195	0.498	0.526	0.169	0.763	0.220	56

55-141	0.094	0.131	0.148	0.119	0.286	0.815	0.207	0.013	0.255	0.002	0.037	0.560	0.217	57
55-138	0.115	0.114	0.100	0.113	0.118	0.205	0.690	0.142	0.402	0.436	0.246	0.795	0.212	58
YT93-159	0.219	0.244	0.247	0.001	0.245	0.138	0.684	0.083	0.365	0.401	0.189	0.692	0.212	59
YCE06-61	0.047	0.062	0.071	0.013	0.557	0.169	0.325	0.238	0.355	0.392	0.275	0.839	0.210	60
91-63	0.238	0.237	0.206	0.019	0.171	0.261	0.474	0.096	0.323	0.361	0.165	0.549	0.206	61
211-8	0.180	0.163	0.128	0.102	0.382	0.021	0.772	0.157	0.435	0.467	0.097	0.618	0.206	62
91-8	0.205	0.170	0.119	0.077	0.310	0.086	0.409	0.263	0.512	0.035	0.356	0.523	0.205	63
55-72	0.354	0.263	0.173	0.002	0.415	0.000	0.860	0.072	0.251	0.294	0.191	0.530	0.201	64
211-137	0.229	0.207	0.162	0.012	0.085	0.038	0.827	0.182	0.454	0.485	0.151	0.518	0.192	65
211-178	0.250	0.259	0.233	0.008	0.153	0.066	0.920	0.072	0.422	0.455	0.058	0.483	0.191	66
55-89	0.064	0.101	0.126	0.048	0.060	0.498	0.626	0.044	0.350	0.387	0.078	0.526	0.190	67
91-76	0.177	0.096	0.041	0.034	0.244	0.020	0.982	0.307	0.384	0.419	0.138	0.607	0.189	68
213-18	0.219	0.151	0.092	0.241	0.788	0.027	0.522	0.006	0.318	0.000	0.006	0.621	0.188	69
91-35	0.088	0.065	0.037	0.033	0.344	0.153	0.766	0.107	0.430	0.463	0.151	0.700	0.187	70
211-259	0.119	0.122	0.106	0.133	0.000	0.008	0.000	0.375	0.538	0.564	0.245	0.345	0.186	71
211-154	0.303	0.246	0.175	0.077	0.096	0.089	0.909	0.025	0.322	0.360	0.010	0.790	0.186	72
211-255	0.015	0.015	0.009	0.174	0.242	0.021	0.533	0.300	0.644	0.664	0.063	0.349	0.185	73
55-54	0.121	0.144	0.144	0.026	0.205	0.004	0.907	0.103	0.589	0.613	0.086	0.500	0.184	74
23-28	0.019	0.029	0.030	0.012	0.451	0.013	0.502	0.114	0.587	0.010	0.593	0.705	0.180	75
55-157	0.075	0.088	0.085	0.018	0.371	0.046	0.977	0.151	0.367	0.403	0.159	0.604	0.180	76
91-71	0.132	0.148	0.142	0.001	0.211	0.194	0.656	0.057	0.431	0.463	0.060	0.560	0.177	77
91-16	0.130	0.102	0.068	0.090	0.175	0.080	0.737	0.061	0.498	0.527	0.041	0.768	0.175	78
55-105	0.116	0.106	0.081	0.052	0.258	0.326	0.572	0.060	0.215	0.260	0.033	0.828	0.173	79
YCE05-164	0.231	0.199	0.161	0.000	0.528	0.117	0.769	0.109	0.162	0.009	0.124	0.401	0.173	80
91-33	0.144	0.156	0.152	0.047	0.116	0.119	0.921	0.070	0.259	0.302	0.126	0.765	0.173	81
91-62	0.081	0.122	0.151	0.037	0.138	0.132	0.615	0.195	0.378	0.012	0.345	0.517	0.171	82
55-45	0.212	0.112	0.048	0.168	0.539	0.006	0.308	0.054	0.248	0.291	0.113	0.360	0.167	83
213-27	0.073	0.066	0.049	0.280	0.129	0.075	0.859	0.109	0.475	0.015	0.077	0.704	0.163	84
55-182	0.231	0.179	0.119	0.018	0.477	0.074	0.708	0.026	0.459	0.003	0.038	0.463	0.156	85
213-19	0.108	0.069	0.033	0.051	0.123	0.048	0.952	0.143	0.429	0.018	0.316	0.530	0.152	86

91-77	0.070	0.065	0.050	0.053	0.127	0.025	0.606	0.119	0.437	0.469	0.138	0.564	0.148	87
213-13	0.067	0.047	0.025	0.144	0.229	0.124	0.627	0.165	0.242	0.285	0.034	0.410	0.147	88
91-36	0.225	0.213	0.173	0.036	0.116	0.075	0.742	0.008	0.233	0.276	0.000	0.553	0.146	89
YCE05-64	0.103	0.101	0.083	0.004	0.188	0.298	0.832	0.038	0.230	0.004	0.048	0.496	0.136	90
213-6	0.021	0.039	0.047	0.227	0.126	0.013	0.459	0.039	0.258	0.300	0.048	0.563	0.125	91
91-21	0.128	0.063	0.019	0.022	0.173	0.211	0.927	0.014	0.307	0.002	0.019	0.633	0.124	92
211-3	0.088	0.030	0.000	0.011	0.292	0.199	0.850	0.021	0.388	0.002	0.019	0.581	0.122	93
55-33	0.197	0.168	0.119	0.019	0.374	0.006	0.907	0.003	0.000	0.000	0.020	0.508	0.119	94
211-61	0.040	0.040	0.030	0.004	0.257	0.067	0.933	0.092	0.293	0.334	0.050	0.000	0.116	95
23-53	0.052	0.052	0.041	0.114	0.364	0.035	0.472	0.007	0.210	0.255	0.007	0.298	0.112	96
211-148	0.055	0.036	0.016	0.008	0.041	0.204	0.655	0.009	0.306	0.345	0.016	0.351	0.109	97
55-164	0.000	0.000	0.027	0.022	0.256	0.042	0.883	0.014	0.432	0.002	0.026	0.801	0.102	98
211-138	0.013	0.016	0.012	0.031	0.029	0.202	0.953	0.040	0.294	0.004	0.035	0.397	0.096	99
91-61	0.051	0.061	0.057	0.017	0.011	0.060	0.937	0.001	0.128	0.000	0.021	0.571	0.078	100
Trait weight	0.087	0.088	0.096	0.151	0.076	0.130	0.029	0.096	0.042	0.069	0.108	0.028		

Note: The integrated D value was calculated from the 12 selected drought-response traits using membership function values and trait weights.
